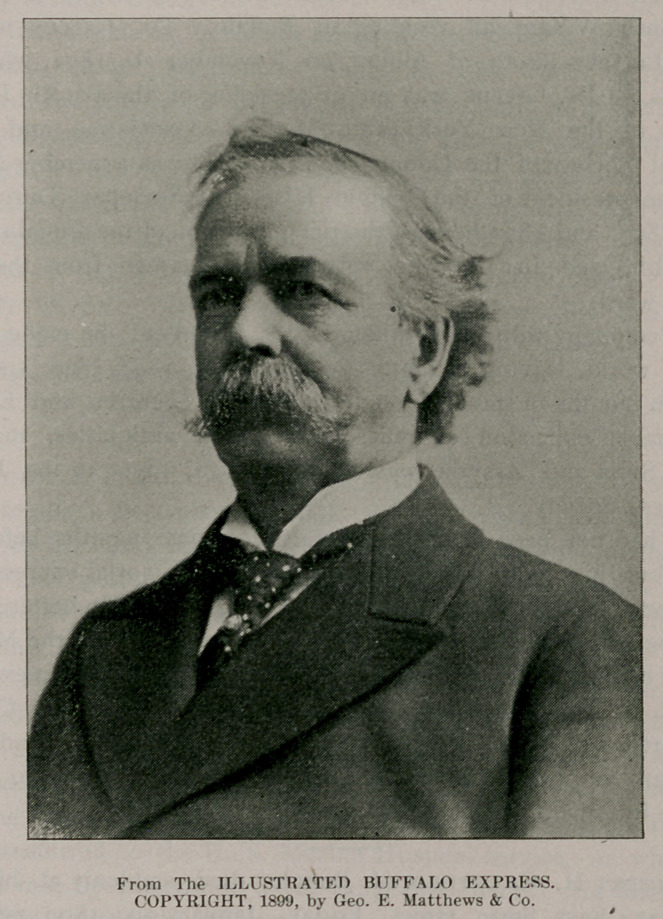# Dr. Joseph C. Greene

**Published:** 1899-02

**Authors:** 


### OBITUARY.

Dr. Joseph C. Greene, of Buffalo, died at his residence, 1125 Main street, Tuesday, January 3, 1899, aged 69 years. He was born at Lincoln, Vt., July 31, 1829, where he continued to reside

From The ILLUSTRATED BUFFALO EXPRESS.
COPYRIGHT, 1899, by Geo. E. Matthews & Co.

during the first sixteen years of his life, attending the public school in winters. In 1846 he entered a boarding school at Nine Partners, Dutchess County, N. Y., and later attended the Barry Academy in Vermont, from which institution he was graduated.

His medical studies were commenced in the office of Dr. Hugh Taggart, a prominent physician of Western Vermont, and were completed by courses in the Woodstock, Castelton and Albany Medical Colleges. He received his degree of M. D. from the Albany school in 1855 and spent the next year in study among the hospitals of New York. He practised for a short time in Charlotte and in 1863 came to Buffalo.

He married Miss Julia Taggart, of Vermont, September 21, 1856. She died in Buffalo in 1882. Three children of this marriage survive, Dr. Dewitt C. Greene, Mrs. Edward Andrews and Mrs. Fred Bush Willard, all residing in Buffalo. Dr. Greene married Mary Burrows Smith, of Albion, on November 26, 1891, who also survives. Dr. Greene was an ex-president of the Fourth District branch of the New York State Medical Association and of the Medical Society of the County of Erie. He was a member and at one time president of the Buffalo Historical Society. During the years 1873 and 1874 he was district physician of the Buffalo Board of Health, and in 1885 he served as alderman from the then second ward.

In company with his brother, Dr. S. S. Greene, he made a tour of the world, leaving Buffalo, September 3, 1888, and spending fourteen months in travel through the eastern countries and Europe. A valuable collection of relics, curios and antiquities, made in Egypt, Syria and Assyria, was, on his return, given to the Buffalo Historical Society.

He had not been in his usual health for some months before his death, but his final sickness was brief. A memorial expressive of the esteem in which the profession held him, couched in better phrase than we can write, is published in the proceedings of the Medical Society of the County of Erie, on page 529. We present an excellent portrait of Dr. Greene, made by the Matthews-Northrup Co., at their art works in this city. His funeral was largely attended by physicians and other prominent citizens, Friday, January 6, 1899, and the interment was at Forest Lawn.

## Figures and Tables

**Figure f1:**